# Application of Weighted Gene Co-Expression Network Analysis to Metabolomic Data from an ApoA-I Knockout Mouse Model

**DOI:** 10.3390/molecules29030694

**Published:** 2024-02-02

**Authors:** Zhe Zhou, Jiao Liu, Jia Liu

**Affiliations:** 1 Institute of Systems Biomedicine, Department of Pathology, School of Basic Medical Sciences, Peking University Health Science Center, Beijing 100191, China; 2 Center of Medical and Health Analysis, Peking University Health Science Center, Beijing 100191, China; 3 Department of Microbiology and Infectious Disease Center, School of Basic Medical Sciences, Peking University Health Science Center, Beijing 100191, China

**Keywords:** weighted gene co-expression network analysis (WGCNA), metabolomics, apoA-I, LC-MS, network

## Abstract

As the ability to collect profiling data in metabolomics increases substantially with the advances in Liquid Chromatography–Mass Spectrometry (LC-MS) instruments, it is urgent to develop new and powerful data analysis approaches to match the big data collected and to extract as much meaningful information as possible from tens of thousands of molecular features. Here, we applied weighted gene co-expression network analysis (WGCNA), an algorithm popularly used in microarray or RNA sequencing, to plasma metabolomic data and demonstrated several advantages of WGCNA over conventional statistical approaches such as principal component analysis (PCA) and partial least squares discriminant analysis (PLS-DA). By using WGCNA, a large number of molecular features were clustered into a few modules to reduce the dimension of a dataset, the impact of phenotypic traits such as diet type and genotype on the plasma metabolome was evaluated quantitatively, and hub metabolites were found based on the network graph. Our work revealed that WGCNA is a very powerful tool to decipher, interpret, and visualize metabolomic datasets.

## 1. Introduction

As an important discipline of systems biology, metabolomics aims to globally understand the metabolic responses of a living system to pathophysiological stimuli or genetic signaling [1,2]. It is an influential approach to search specific biomarkers and to identify disturbed pathways in both basic and clinical research [3,4,5]. The metabolome, the subject of metabolomics, is defined as all the metabolites/chemicals with a molecular weight < 1500 Da [6]. With the advent of more and more advanced technologies and instruments, we are now capable of collecting tens of thousands of data in a high-throughput manner. Traditional statistical analysis methods/algorithms have several limitations in treating this increasing flood of data. First, conventional univariate statistical methods concerning fold change and *p*-values are biased to metabolites with high changes in expression, while lacking the consideration of the relationship between each pair of metabolites as a whole. Second, although commonly used multivariate statistical approaches such as hierarchical cluster analysis (HCA), principal component analysis (PCA), partial least squares discrimination analysis (PLS-DA) and orthogonal partial least squares discrimination analysis (OPLS-DA) treat all molecular features in a holistic manner, they cannot relate molecular features to phenotypes quantitatively [7,8]. Thus, new approaches, with the ability to both extract meaningful information from multi-dimensional datasets effectively and be understood by users easily, are needed urgently.

Weighted gene co-expression network analysis (WGCNA) is a popular and powerful data analysis tool that could be used to construct networks, generate modules and identify central players in a module [9,10]. Although WGCNA was initially developed for the analysis of microarrays or RNA-seq datasets, the concept and strategy of WGCNA in data analysis can also be applied to proteomics and metabolomics datasets whose formats are similar to that of microarrays [11]. A WGCNA strategy is as follows: 1. Construct a network based on the co-expression similarity between each pair of genes, thus the connection between genes can be obtained. 2. Merge a large number of genes into a small number of modules by hierarchical clustering, simplifying and facilitating the following analysis. 3. Relate modules assigned to phenotypic traits, by means of which the genes and pathways associated with the phenotypic traits can be found. 4. Study inter-module relationships by module eigengene (ME) correlation clustering, and phenotypic traits can also be added to examine the relationships between phenotypic traits and modules. 5. Find hub drivers/players in the module of interest: the node with the highest connectivity and the largest number of connections is the most important player in this module. This function provides greater value in the field of metabolomics. Unidentified or poorly annotated molecular features, which are the main bottleneck in metabolomics [12,13], can be inferred by their identified neighbors.

As far as we know, there are few studies on the feasibility, adaptability and advantages of the application of WGCNA to metabolomics datasets derived from KO mice [14]. In this study, we apply WGCNA to plasma metabolomic data to explore the impact of diet type and genotype on the plasma metabolome in apoA-I (−/−) mice. We compared the power and abilities of traditional data analysis methods and WGCNA in the visualization, interpretation and exploration of our data. Our work demonstrated that WGCNA is a useful and valuable tool for metabolomic data analysis.

## 2. Results and Discussion

### 2.1. Non-Targeted LC-MS Metabolomics Data of ApoA-I-Knockout Mice

We chose the annotated dataset of the work of Lemin Zheng et al. [15]. In their work, metabolomic and lipidomic profiles of plasma from apoA-I-knockout and control mice fed a high-fat diet (HFD) or chow were obtained by LC-MS, and the data were analyzed by a conventional method. As a proof-of-principle study, we used metabolomic data from apoA-I-knockout mice and control mice at the time point of 8 weeks of feeding for WGCNA analysis. There were 6 mice in each group except 5 mice in apoA-I knockout mice at 8 weeks’ chow. Compounds were identified by MSDIAL using its built-in database, and the abundance of each molecular feature was characterized by its peak area. This produced a list of 4111 molecular features, although a large number of these molecular features remain unidentified. Compared with other solutions usually used in the field of metabolomics, WGCNA offers much more information and provides a better understanding of the big data obtained, vide infra.

### 2.2. Weighted Co-Expression Network Construction

**Figure 1:**
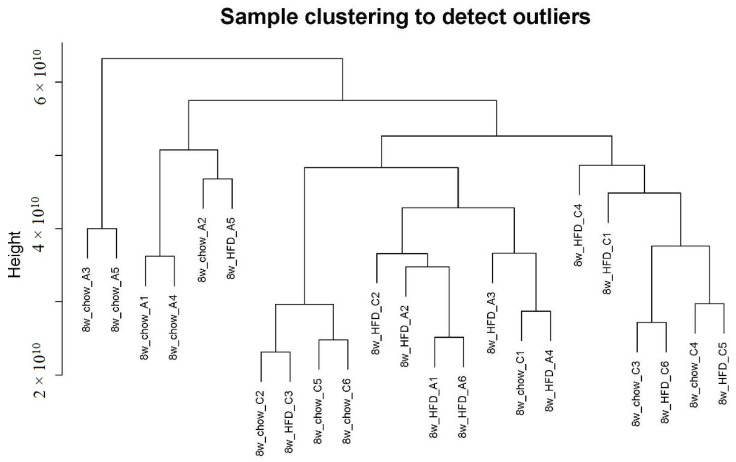
Sample clustering plot. No outlier was observed.

**Figure 2:**
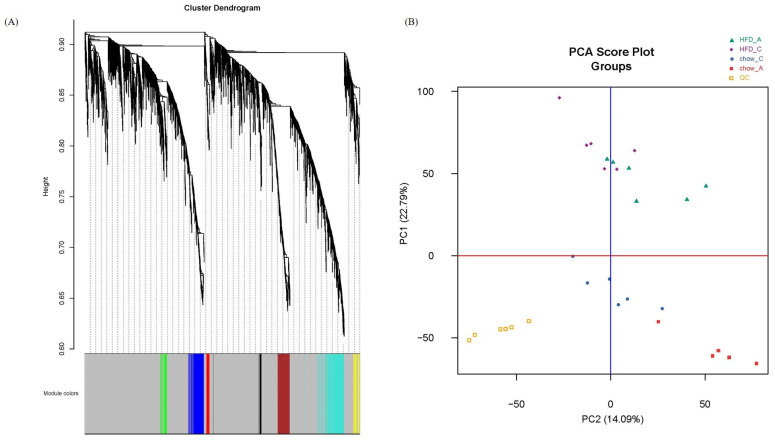
(**A**) Cluster dendrogram and module assignment. Metabolites were clustered based on their distances. A total of 4111 metabolites were assigned to 8 modules including grey module. (**B**) PCA score plot of metabolome from apoA-I-knockout and control mice fed HFD for 8 weeks and pooled QCs.

Gene co-expression analysis is a data analysis technique that helps identify groups of genes/metabolites with similar expression patterns across several different conditions [16]. We first used the R package WGCNA to draw a sample tree plot to check whether there were outlying samples. As shown in the sample tree in Figure 1, no outlier was observed, indicating the consistency of samples in treatment and instrument analysis. To reach the scale-free topology, β = 4 was considered in the following study; see Figure S1 in Supplementary Material for details. Module assignment in WGCNA is a tunable process that permits users to set several parameters to influence the constructed network. Here, we constructed a signed network with minKMEtoStay = 0.7. A total of 4111 metabolites were then assigned to 8 different metabolite modules, and each module was visualized by a unique color, as shown in Figure 2A. The metabolites that failed to be assigned to any modules were placed in the grey module, which was disregarded and not considered in the following analysis. There were 305 metabolites in the turquoise module, 207 metabolites in the blue module, 183 metabolites in the brown module, 59 metabolites in the yellow module, 51 metabolites in the green module, 41 metabolites in the red module and 35 metabolites in the black module. The weighted network could be also visualized in a heatmap plot, as displayed in Figure S2 in the Supplementary Materials. The module eigengenes/eigenmetabolites (MEs) of all samples are listed in Supplementary Table S1. Clustering molecular features into a few modules could reduce the dimension of a dataset, prevent multiple testing problems [17] and allow phenotypic traits to be related to several variables, such as ME, instead of tens of thousands of individual molecular features, vide infra.

Traditionally, metabolomics data were analyzed by principal component analysis (PCA) in a global and unbiased fashion. PCA score plots could tell us the relative similarity of all samples along the first several principal components, while PCA loading plots allowed users to find the metabolites that contribute the most to group differentiation. However, PCA is not powerful and effective in terms of fine classifying and hierarchical clustering [18]. For instance, Figure 2B shows the PCA score plot of our data. Principal component 1 explained 22.79% of the variation in the data. The mice fed with HFD are located mainly in the upper part of the plot, while mice fed with chow are situated in the lower part. Both principal component 1 and principal component 2 (which explained 14.09% of the variation) were associated with the genotype. However, PCA can neither provide information in more detail nor relate molecular features to phenotypes quantitatively. It is also the same for PLS-DA and OPLS-DA.

### 2.3. Association of Modules to Phenotype and Module–Module Relationship

**Figure 3:**
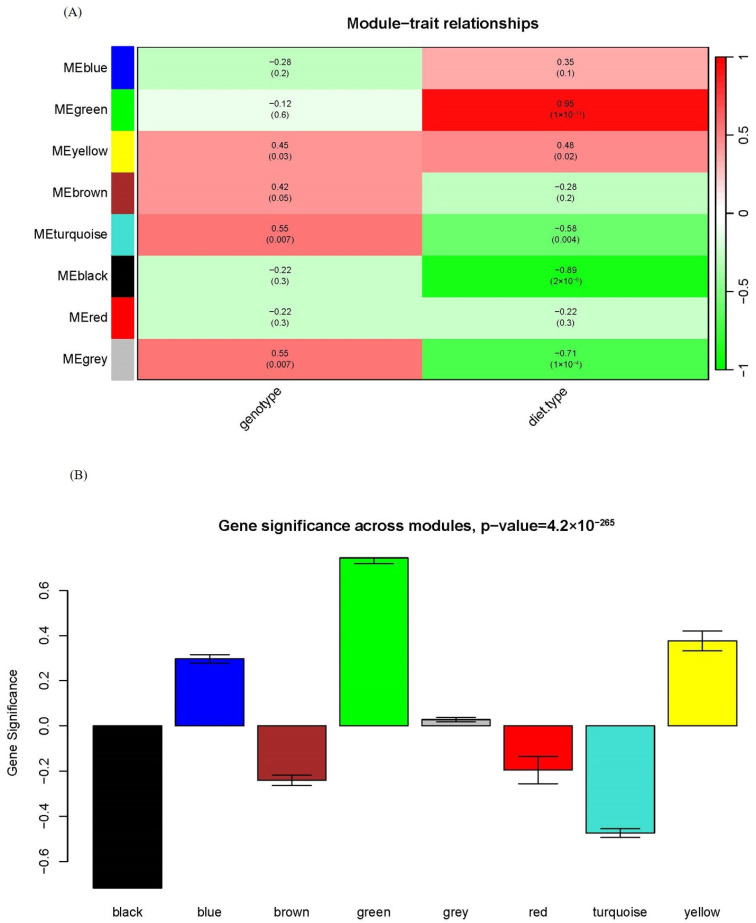
Module–trait and module–module relationships of the network. (**A**) Module–trait relationship. Each row corresponds to a module eigengene and each column to a trait. Each cell contains the corresponding correlation and *p*-value. The table is color-coded by correlation according to the color legend. (**B**) Bar plot of module significance defined as the mean gene/metabolite significance across all genes/metabolites in the module. The green and black modules had the highest relevance to diet type.

One of the advantages of WGCNA over other statistical approaches usually used in metabolomics is that WGCNA can associate specific modules with clinical traits. We assessed the relationship between each module and genotype/diet type in Figure 3A. While Zheng et al. claimed that there were more differences between HFD and chow feeding than between apoA-I-knockout mice and control mice in their previous study [15], we could draw this conclusion in a more quantitative way. Only two modules displayed significant associations with genotype: yellow and turquoise. In contrast, four modules showed significant associations with diet type: green, yellow, turquoise and black. Among them, the green module was strongly and positively correlated with diet type and the black module was strongly and negatively correlated with diet type, with the corresponding correlation and *p*-value between module eigengene and diet type being 0.95 and 1 × 10^−11^ for the green module and −0.89 and 2 × 10^−8^ for the black module; see Figure 3A for detail. It indicated that the green and black modules have a profound biological role as HFD stimuli.

Another way to associate modules with phenotype is module significance (MS), which is the mean gene/metabolite significance in a given module. Figure 3B compared the MS with diet type among all modules, and the green and black modules had the highest relevance to diet type in line with the module–trait association in Figure 3A. These results indicated that WGCNA could identify biologically relevant modules and metabolites, facilitating further biological investigations.

**Figure 4:**
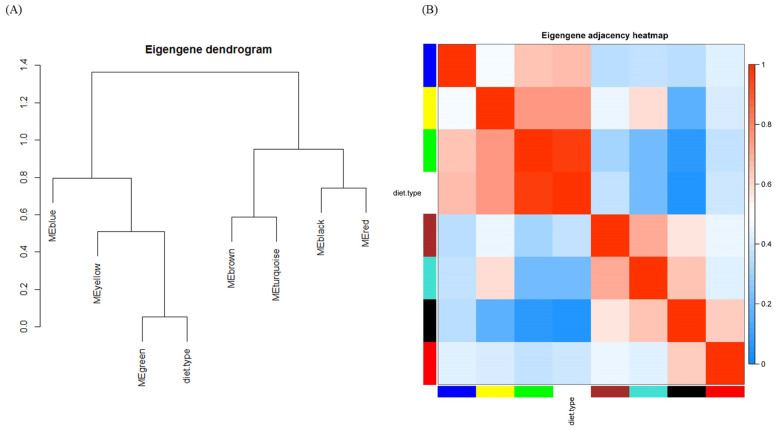
Eigengene network indicating the relationships among modules and diet type. (**A**) Eigengene dendrogram and (**B**) heat map.

The module–module relationship is illustrated as a dendrogram (Figure 4A) and a heatmap (Figure 4B). To be more informative, diet type was also interposed among modules. Consistent with Figure 3, the green module was highly related to diet type. Beyond Figure 3, the distances and relationships among modules were unveiled, e.g., the meta-module of the green module and diet type were negatively related to the black module, as expected. The correlation matrix graph and scatter diagram of module eigengenes are shown in Supplementary Material Figure S3.

### 2.4. Intramodular Analysis and Hub Metabolites

**Figure 5:**
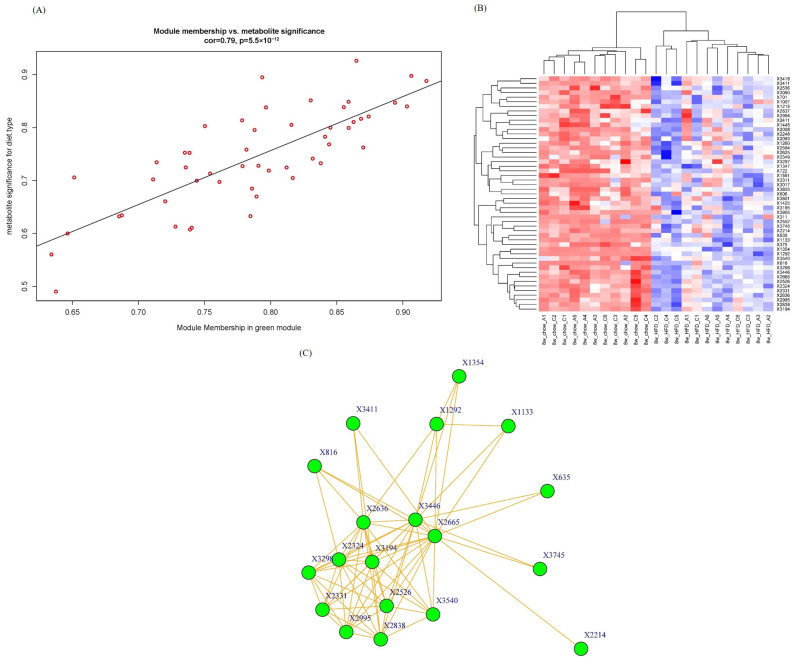
(**A**) A scatterplot of GS for diet type vs. MM in the green module. Red circles stand for metabolites in the green module. There is a highly significant correlation in this module; (**B**) Metabolite abundance heatmap of green module. Red color means up-regulation of metabolites and blue color means down-regulation; (**C**) Network graph of green module. Igraph package (version 1.5.1) was used for drawing.

As the green module has the highest significance with phenotypic traits, we pay more attention to this module for the intramodular analysis and hub metabolite studies. We calculate the module membership (MM) and gene/metabolite significance (GS) for each metabolite in the green module and plot them in a scatter diagram in Figure 5A. A high correlation (0.79) was observed between GS and MM of metabolites in the green module. The higher the GS and MM of a metabolite, the greater the importance of this metabolite to diet type.

The metabolite abundance heatmap of the green module is illustrated in Figure 5B. The rows and columns represent metabolites and samples, respectively. The metabolomic profiling in the green module successfully differentiated mouse groups fed with HFD and chow, indicating that these metabolites were deeply involved in lipid metabolism progression.

Network graph is also a powerful tool in WGCNA to pinpoint the key and hub genes/metabolites within a module [19]. To seek and find hub metabolites in the green module, a network graph was plotted in node and edge mode in Figure 5C. Nodes stand for metabolites, and edges stand for connection strengths. The connection strengths between each pair of metabolites, characterized by TOMs, ranged from 0.0093 to 0.2032, with an average of 0.062 ± 0.037. Only strong connections (TOM > 0.13) were drawn in the network. Generally speaking, the stronger connections a metabolite has, the more important role it serves in biological progress because a lot of other metabolites change as this hub metabolite changes. In this case, hub metabolite X816, with a retention time of 10.44 min and an *m*/*z* of 165.0587, was upregulated in the HFD group. X816 was reasonably identified as 2-coumaric acid, which is a hydroxyl derivative of cinnamic acid. Recently, p-coumaric acid, another hydroxyl derivative of cinnamic acid, has been found to have multiple biological activities including antioxidant, anti-cancer and anti-arthritis activities [20]. And a study by Abdel-Moneim A proved that p-coumaric acid could inhibit type 2 diabetes-induced neurodegeneration in a rat model [21]. Several potential explanations can be considered. The absence of apoA-I could result in impaired hepatic clearance mechanisms, leading to the accumulation of 2-coumaric acid. And apoA-I plays a crucial role in modulating the gut microbiota composition and function, and its deficiency might influence the gut microbial metabolism of dietary components, including 2-coumaric acid. The physiological functions of X816 and the enzymes and genes involved deserve further investigation. This network graph is very useful in metabolomics analysis because it is helpful to annotate and infer unidentified molecular features (the main bottleneck in metabolomics) by the identified nodes/metabolites strongly connected to them.

## 3. Methods

### 3.1. Data

The annotated dataset comes from the published work of Lemin Zheng et al. [15]. ApoA-I-knockout mice and control mice at the time point of 8 weeks of feeding were selected for the following analysis. All mice were male. There were four groups: 6 ApoA-I-knockout mice fed HFD, 5 ApoA-I-knockout mice fed chow, 6 control mice fed HFD and 6 control mice fed chow. The raw data were first preprocessed by Mass Spectrometry–Data Independent AnaLysis software (MSDIAL, version 3.52) [22]. After peak extraction, the exported data were imported into R (version 3.4.4) for further analysis. There were 23 samples and 4111 metabolites in the dataset.

### 3.2. Statistical Analysis

Most of the data analyses were conducted in R (version 3.4.4).

#### 3.2.1. WGCNA Network Construction and Module Detection

In data preprocessing, a sample tree plot was drawn to check whether there were outliers. After that, network construction was performed following the pipelines of WGCNA [9]. Metabolites were clustered by their similarity (distance), which was calculated by the correlation coefficient between two metabolites. A metabolite module is a cluster of highly co-expressed metabolites. The power/soft threshold was determined as the lowest power for which the R^2^ reaches 0.90. The critical parameters in network construction were as follows: power = 4, networkType = ‘signed’, minKMEtoStay = 0.7. MinKMEtoStay was set to 0.7 to cluster highly correlated metabolites into a module and to simplify further analysis. Other parameters were at their default values.

WGCNA exploits hierarchical clustering to determine metabolite modules, and each module was assigned to a unique color. The metabolites that could not be assigned to any of the modules were placed in a grey module. The module eigengene/eigenmetabolite (ME) of a module is defined as the first principal component of the module and indicates the global expression level of the module. The correlations among modules were studied by hierarchical clustering of the MEs of each module.

#### 3.2.2. Association of Modules with Phenotypes and Module–Module Relationships

WGCNA exploits the module eigengene, the first principal component of a given module, and the module significance (MS), the average gene/metabolite significance (GS) of all genes/metabolites in the module, to find the modules highly related to the phenotype of interest. Hierarchical clustering and heat maps of MEs were performed to view the relationships among modules.

### 3.3. Visualization

All figures were plotted by R (version 3.4.4). PCA was performed with the R package metabolomics. A network was created with the R package igraph, and only strong connections (Topological Overlap Measure, TOM > 0.13) were drawn in the network.

## 4. Conclusions

In this article, we applied the strategy of WGCNA, a popular and powerful data analysis tool in the RNA-seq database, to metabolomics data as a proof of principle. We chose the dataset of a published work concerning the metabolomic profiles of plasma from apoA-I-knockout and control mice fed with different diet types. We demonstrated several advantages of WGCNA over traditional approaches. Tens of thousands of molecular features are assigned to a limited number of modules, facilitating the following analysis greatly. Clinical traits, whether continuous variables or classified variables, could be associated with modules quantitatively. In particular, the green and black modules were strongly associated with diet type, which deserves further attention. Hub metabolites such as 2-coumaric acid were found to be susceptible to HFD stimuli based on the network graph. Moreover, the network graph was helpful in annotating unidentified metabolites by their identified neighbors. Our work demonstrated that WGCNA is a useful and valuable tool for metabolomic data analysis.

## Data Availability

Data are available on request from the authors. Data were obtained from Lemin Zheng and are available with the permission of him.

## Supplementary Materials

The following supporting information can be downloaded at https://www.mdpi.com/article/10.3390/molecules29030694/s1, Figure S1: Selection of the soft-thresholding powers; Figure S2: Heat map plot of the metabolites network; Figure S3: Correlation matrix graph of module eigengene; Table S1: ME of all samples.

## Abbreviations

WGCNA	weighted gene co-expression network analysis
TOM	Topological Overlap Measure
HFD	high-fat diet
MS	module significant
MM	module membership
PCA	principal component analysis
