# Supplementary material for: Application of Weighted Gene Co-Expression Network Analysis to Metabolomic Data from an ApoA-I Knockout Mouse Model
Source: Molecules. 2024 Feb 2;29(3):694. doi: 10.3390/molecules29030694 (PMC10856800; doi:10.3390/molecules29030694)
Supplement: Supplementary file 1 [file molecules-29-00694-s001.zip › molecules-2781303-Supplementary material.pdf]

Application of Weighted Gene Co-expression Network Analysis to Metabolomic Data from an ApoA-I Knockout Mouse Model

Zhe Zhou[1] Jiao Liu[2*] and Jia Liu[3]*

[1] Institute of Systems Biomedicine, Department of Pathology, School of Basic Medical Sciences, Peking University Health Science Center, Beijing, 100191, China

[2] Center of Medical and Health Analysis, Peking University Health Science Center, Beijing, China.

[3] Department of Microbiology and Infectious Disease Center, School of Basic Medical Sciences, Peking University Health Science Center, Beijing, China

*** Corresponding author contact information**

Jiao Liu, Ph.D.

E-mail: liujiao@bjmu.edu.cn

Jia Liu, Ph.D.

E-mail: liujia894@bjmu.edu.cn

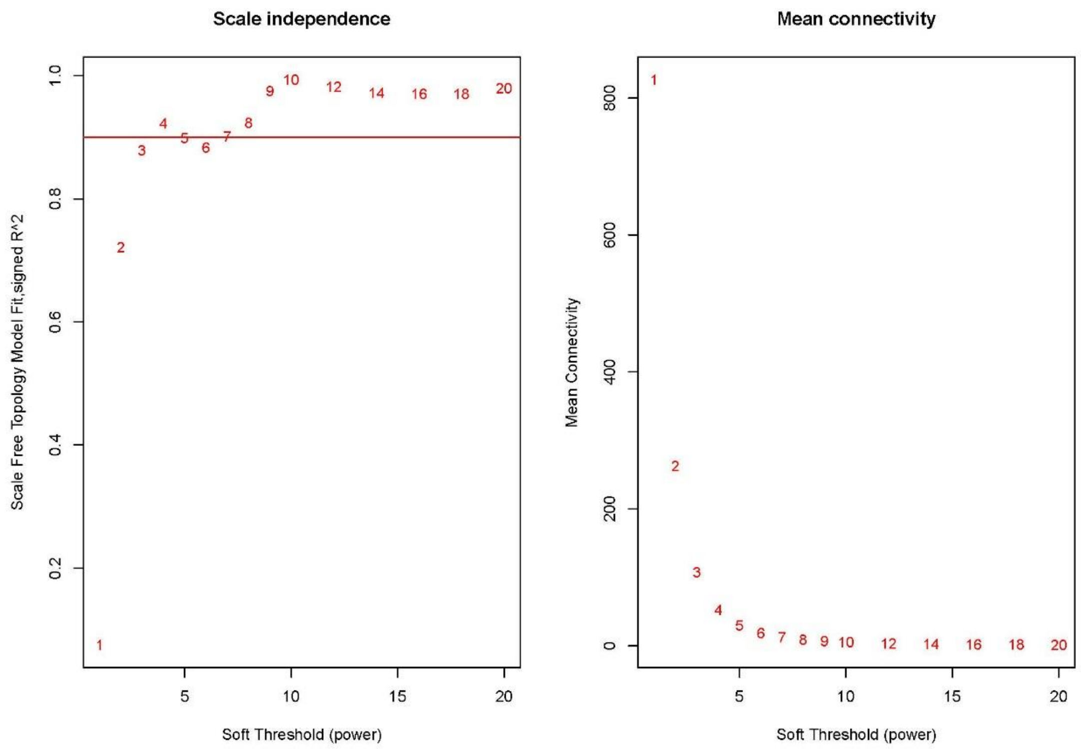

Figure S1. Selection of the soft-thresholding powers. The left panel shows the scale-free fit index versus soft-thresholding power. The right panel displays the mean connectivity versus soft-thresholding power. Power 4 was chosen, for which the fit index curve flattens out upon reaching a high value (> 0.9).

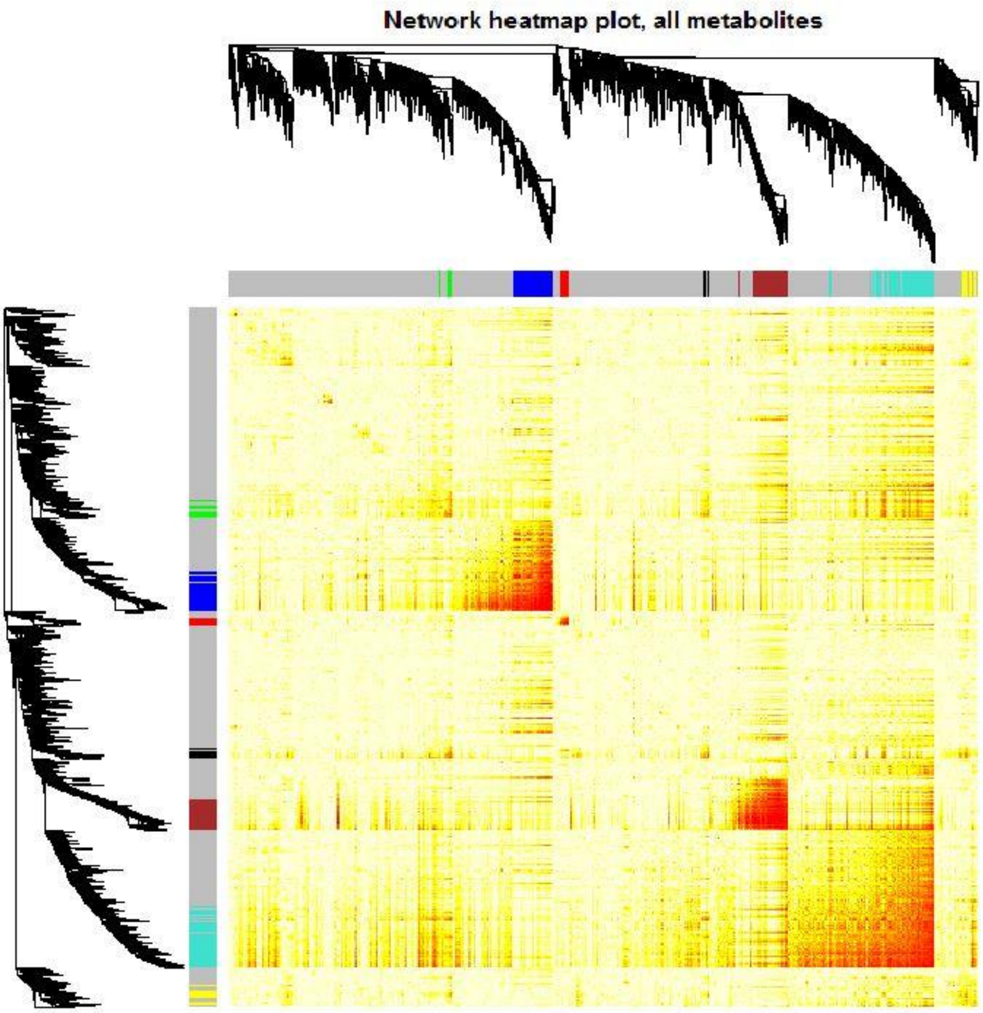

Figure S2. Heat map plot of the metabolites network. The heatmap depicts the Topological Overlap Matrix (TOM) among all metabolites in the analysis. Light color represents low correlation and progressively darker red color represents higher correlation. Blocks of darker colors along the diagonal are the modules. The metabolites dendrogram and module assignment are also shown along the left side and the top.

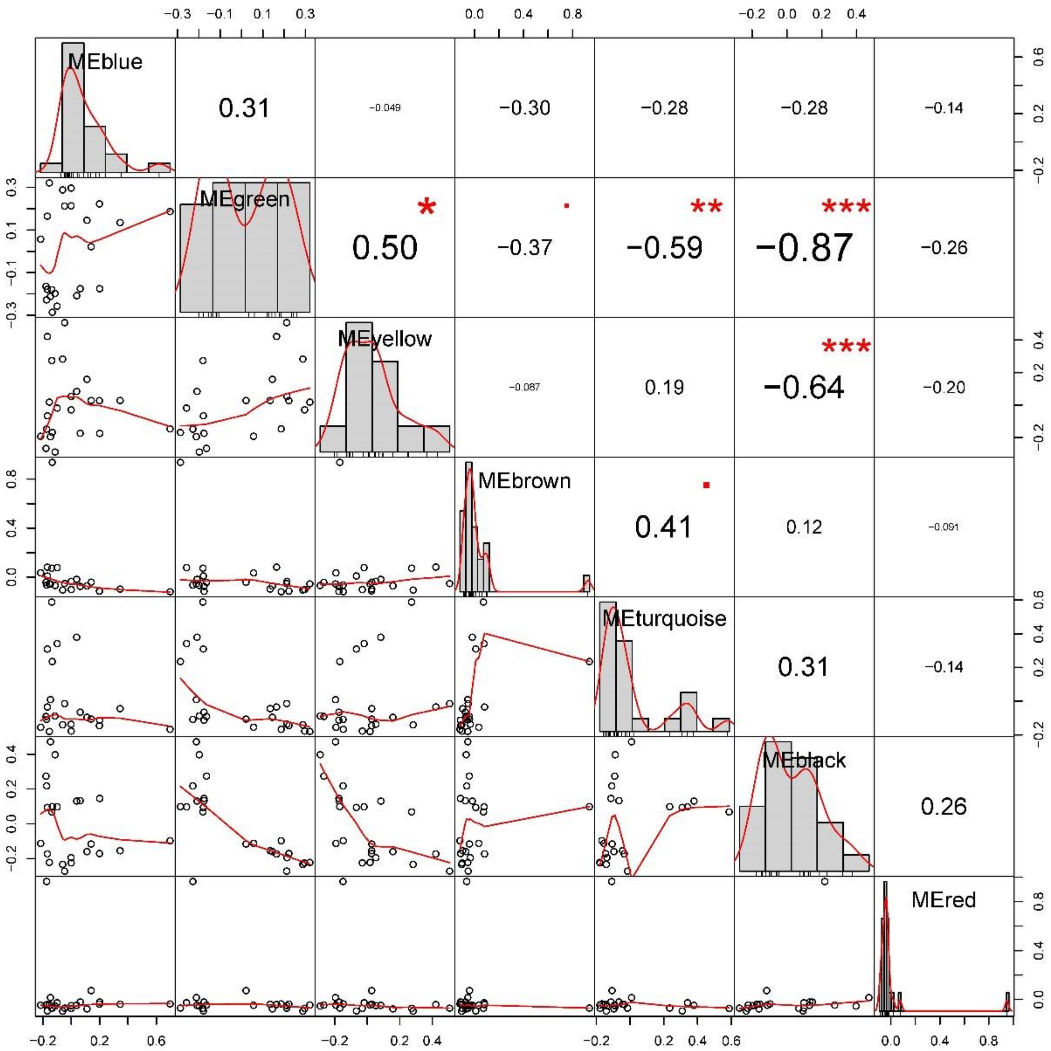

Figure S3. Correlation matrix graph of module eigengene. The significance of correlation is indicated by the digit size. Module black and module green are inversely correlated as expected.
